# CAR Beyond αβ T Cells: Unleashing NK Cells, Macrophages, and γδ T Lymphocytes Against Solid Tumors

**DOI:** 10.3390/vaccines13060654

**Published:** 2025-06-19

**Authors:** Yunjia Xian, Lu Wen

**Affiliations:** 1 Cancer Center, Union Hospital, Tongji Medical College, Huazhong University of Science and Technology, Wuhan 430022, China; u202113677@hust.edu.cn; 2 Tongji Medical College, Huazhong University of Science and Technology, Wuhan 430022, China; 3 Hubei Key Laboratory of Precision Radiation Oncology, Wuhan 430022, China; 4 Institute of Radiation Oncology, Union Hospital, Tongji Medical College, Huazhong University of Science and Technology, Wuhan 430022, China

**Keywords:** CAR-T, CAR-NK, CAR-M, CAR-γδ T, cell therapy, cancer immunotherapy, solid tumor

## Abstract

Chimeric antigen receptor (CAR)-engineered cell therapy represents a landmark advancement in cancer immunotherapy. While αβ CAR-T therapy has demonstrated remarkable success in hematological malignancies, its efficacy in solid tumors remains constrained mainly by factors such as antigen heterogeneity, immunosuppressive microenvironments, and on-target/off-tumor toxicity. To overcome these limitations, emerging CAR platforms that utilize alternative immune effectors, including natural killer (NK) cells, macrophages, and γδ T lymphocytes, are rapidly gaining traction. This review systematically analyzes the mechanistic advantages of CAR-NK, CAR-M, and CAR-γδ T cell therapies, while critically evaluating persistent challenges in clinical translation, including limited cell persistence, manufacturing scalability, and dynamic immune evasion mechanisms. We further discuss innovative strategies to enhance therapeutic efficacy through some viable strategies. By bridging fundamental immunology with translational engineering, this work provides a roadmap for developing CAR therapies capable of addressing the complexities of solid tumor eradication.

## 1. Introduction

Cancer imposes a devastating toll on global health, remaining a central focus of medical research. Alongside conventional modalities like surgery, radiotherapy, and chemotherapy, cancer immunotherapy (CIT), which activates the immune system, has emerged as the fourth pillar of cancer treatment, driving transformative advances in oncology. The most clinically impactful CIT strategies include adoptive cell therapy (ACT) and immune checkpoint inhibitors (ICIs). Early ACT approaches developed in the 20th century relied on isolating autologous peripheral lymphocytes or tumor-infiltrating lymphocytes (TILs), expanding them ex vivo, and reinfusing them into patients. However, these methods showed limited efficacy, primarily in melanoma. Subsequent breakthroughs arose with genetic engineering technologies that enable the ex vivo construction of CARs and generation of CAR-T cells, dramatically expanding the therapeutic potential of ACT across diverse malignancies.

CAR-T cell therapy, as a paradigm-shifting modality, has achieved unprecedented success in hematological malignancies by redirecting αβ T cells to eradicate CD19^+^ B-cell malignancies, such as acute lymphoblastic leukemia(ALL) and adult high-grade B-cell lymphoma [1]. Unlike hematological cancers, solid tumors employ metabolic competition, stromal barriers, and immune checkpoint networks to limit T cell efficacy. The translation of this success to solid tumors has been hampered by multifaceted barriers, including antigen heterogeneity, immunosuppressive tumor microenvironments (TME), and acute toxicities.

Recent research has shifted focus to alternative immune effectors, such as natural killer (NK) cells, macrophages, and γδ T lymphocytes, leveraging their inherent biological advantages to overcome existing limitations. This review systematically analyzes the unique biological advantages of these therapies, evaluates the developmental prospects of novel CAR-based approaches beyond αβ T cell limitations, and investigates the current clinical status, persistent challenges, and potential solutions in their application for solid tumor management.

## 2. The Evolution of CAR Cell Therapy

**Figure 1:**
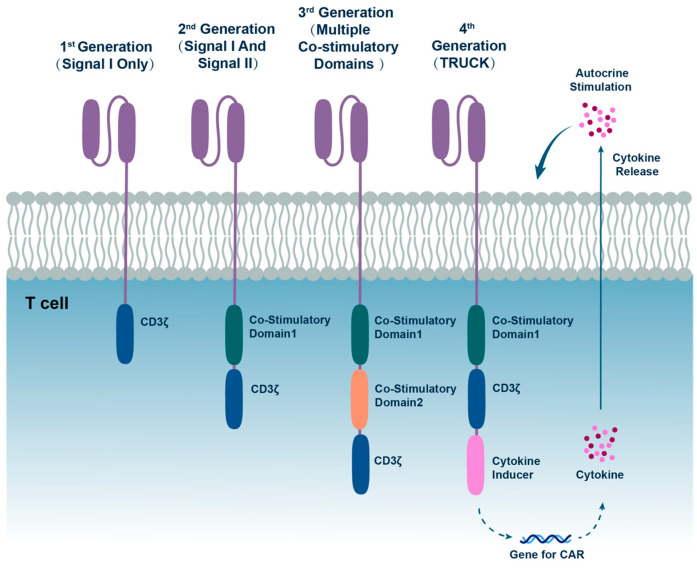
The CAR structures of different generations. The basic structure of CAR includes four parts: an extracellular antigen-binding domain, a hinge region, a transmembrane structural domain, and an intracellular signaling domain. The improvements in each generation mainly focus on the design of the intracellular signaling domain. The first-generation CAR relies solely on CD3ζ to provide the primary signal for T cell activation. The second-generation CAR can provide both primary and secondary signals. On this basis, the third-generation CAR introduces additional costimulatory domains. The fourth-generation CAR, also known as TRUCK, incorporates a structure that induces the secretion of cytokines such as IL-12. These cytokines can modulate the tumor microenvironment, enhance the activity of CAR cells, and recruit a second wave of immune cells.

CAR is a modular synthetic receptor whose core structure consists of four parts: an extracellular antigen-binding domain, a hinge region, a transmembrane structural domain, and an intracellular signaling domain. As the core driver module of CAR function, the design of the intracellular signaling domain directly determines the direction of technological iteration (Figure 1, Table 1). The first generation of CARs only contains primary activation signals (Signal I) provided by CD3ζ. The second generation integrates co-stimulatory signals such as CD28 or 4-1BB on top of this (Signal II), which significantly enhances T-cell expansion capacity and persistence. While third-generation CAR constructs incorporate multiple co-stimulatory domains to enhance signaling, comparative preclinical and clinical analyses have not conclusively demonstrated their superiority over second-generation CAR architectures in therapeutic efficacy or persistence. The fourth generation CAR (TRUCK), on the other hand, remodels the tumor microenvironment through the secretion of cytokines such as IL-12, and has shown positive results in solid tumors by enhancing T-cell activation and recruiting a second wave of immune cells toward the tumor site [2,3]. The current study focuses on the optimization of co-stimulatory domain combination strategies and their adaptation in the solid tumor microenvironment, as well as exploring the mechanism of the synergistic effect of transmembrane domains and signaling domains on CAR-T function.

## 3. Therapeutic Landscape of CAR-NK Therapy

### 3.1. The Immune Function of NK Cell

NK cells are pivotal components of the innate immune system and have garnered significant attention as the most frequently utilized immune cells in CAR therapies other than αβ T cells. Unlike T and B cells, NK cells integrate activation and inhibitory signals to determine whether to eliminate the target cell. The primary activating receptors on the NK cell surface include NKG2D, NKp44, NKp46, and NKp30. Conversely, the main inhibitory receptors are the killer cell Ig-like receptors (KIRs) and CD94/NKG2A, which predominantly transmit inhibitory signals via recognition of major histocompatibility complex (MHC) class I molecules on target cells [10,11,12].

**Figure 2:**
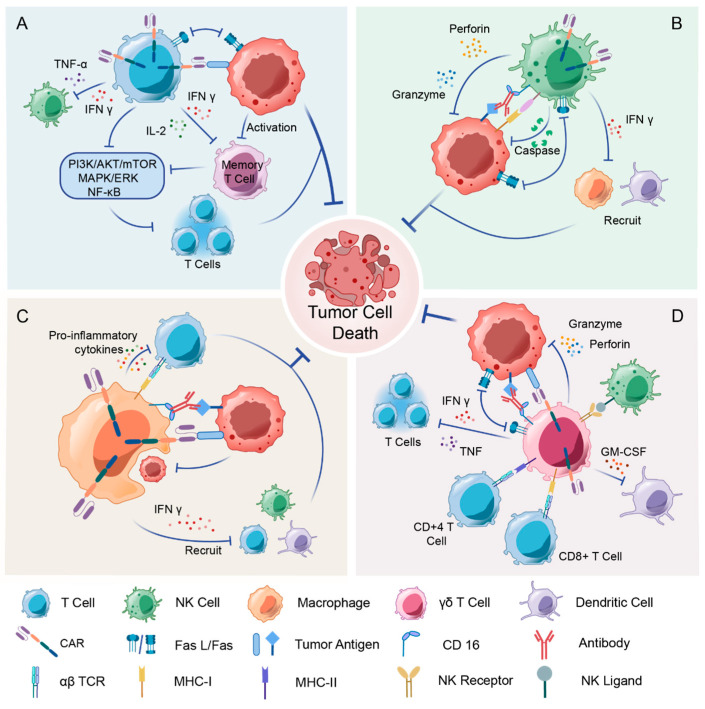
Mechanisms of CAR-engineered immune cells against solid tumors. (**A**) CAR-T cells directly eliminate tumors by engaging surface antigens through their chimeric receptors. This triggers the release of perforin/granzyme and Fas/FasL-mediated apoptosis. However, they face challenges in immunosuppressive microenvironments. (**B**) CAR-NK cells, on the other hand, use innate killing mechanisms via granzyme/perforin and TRAIL pathways. They also work together with CAR targeting and antibody-dependent cellular cytotoxicity (ADCC) through CD16. (**C**) CAR-M have a dual role: they phagocytose antigen-expressing cancer cells and reprogram into pro-inflammatory M1 phenotypes to secrete IL-12 and TNF-α. This dismantles stromal barriers and recruits adaptive immunity. (**D**) CAR-γδ T cells are unique in that they integrate CAR specificity with γδ TCR-mediated stress antigen recognition, enabling MHC-unrestricted killing via NKG2D ligands while acting as antigen-presenting cells to amplify αβ T cell responses. These engineered cells overcome traditional CAR-T limitations through complementary mechanisms: innate adaptability, microenvironment remodeling, and dual-targeting strategies.

The “missing self” hypothesis posits that, since most normal cells express MHC class I molecules on their surface, NK cells become activated upon encountering a target cell with deficient or downregulated MHC class I molecules, thereby resulting in diminished inhibitory signaling [13]. Upon activation, NK cells induce apoptosis in tumor cells primarily through direct killing mechanisms, such as the release of perforin and granzymes, as well as the secretion of members of the tumor necrosis factor (TNF) family, including the expression of Fas ligand (FasL) or TNF-related apoptosis-inducing ligand. Additionally, NK cells can eliminate target cells through antibody-dependent cellular cytotoxicity (ADCC) mediated by the Fc receptor CD16 [14] (Figure 2).

### 3.2. Advantages of CAR-NK Cells over CAR-T Cell Solid Tumors

In the context of solid tumor therapy, CAR-natural killer (NK) cell therapy has emerged as a superior alternative to CAR-T cell therapy, offering several distinct advantages. NK cells, unlike T cells, exhibit a rapid response to tumor recognition and do not require activation or differentiation [15]. One of the characteristics of solid tumors is antigenic heterogeneity, which prevents CAR-T cells from killing tumor cells that lack CAR-targeted antigens on their surface. In contrast, CAR-NK cells are able to mediate target cell killing via both CAR and innate NK cytotoxicity, improving the killing efficiency. Preliminary evidence of the effectiveness of second- and third-generation CAR-NK in preclinical work against a range of antigens and cell types has been demonstrated, including glioblastoma [16], breast cancer [17], ovarian cancer [18], and pancreatic cancer [19], among others.

CAR-NK therapy also boasts a safety profile that is unparalleled by CAR-T therapy. Conventional CAR-T therapy typically involves the collection and genetic modification of autologous T cells, a process that is often time-consuming and inefficient. Moreover, allogeneic transplantation of CAR-T cells can lead to severe graft-versus-host disease (GVHD). In contrast, NK cells are not restricted by MHC molecules, and therefore, they do not induce GVHD [20]. This feature is particularly significant, considering that the cytotoxicity of patients’ own NK cells is often diminished after chemotherapy, necessitating allogeneic transplantation in most CAR-NK therapy scenarios. Moreover, CAR-T cells are highly susceptible to inducing cytokine release syndrome (CRS) through the substantial release of TNF-α, IL-1β, and IL-6 upon targeting tumor antigens. CAR-NK cells initiate anti-tumor activity through direct cell death. The release of neoantigens and the production of dominantly produced IFN-γ following target cell lysis do not trigger CRS [21]. Moreover, CAR-T cells may cause “on-target/off-tumor toxicity” due to the lack of tumor-specific antigens [22].

**Figure 3:**
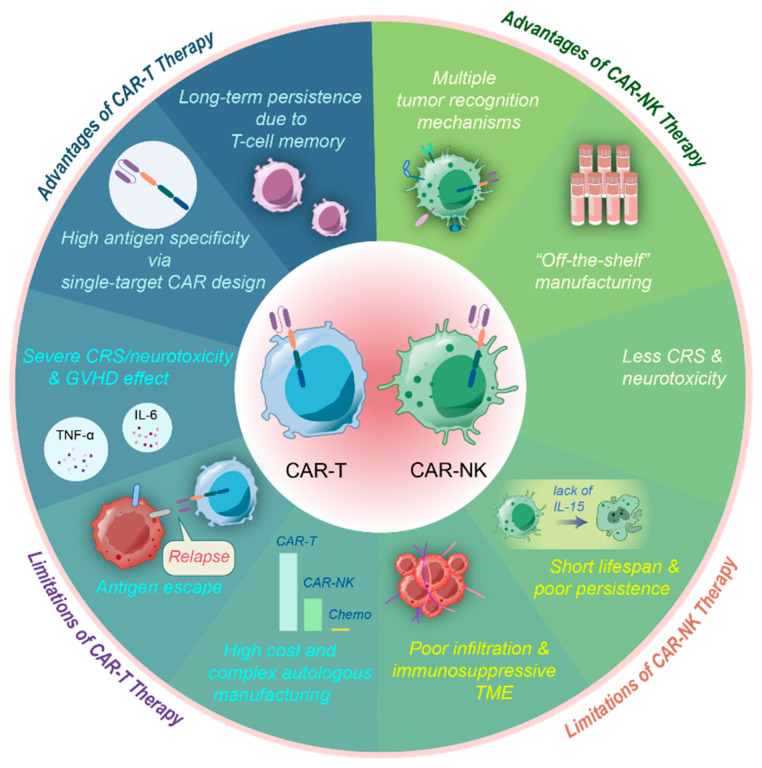
Advantages and limitations of CAR-T and CAR-NK cells. CAR-T and CAR-NK therapies each have distinct advantages and limitations. Although CAR-T cell research is more established and has demonstrated efficacy in hematological malignancies and metastatic melanoma, its application in non-melanoma solid tumors is limited due to immunosuppressive TME, antigen heterogeneity, poor T cell infiltration, and severe adverse effects such as cytokine release syndrome (CRS) and graft-versus-host disease (GVHD). Additionally, CAR-T cell preparation is costly and time-consuming, and patients often have poor tolerance. In contrast, CAR-NK cells show significant potential in treating solid tumors, addressing many of the challenges faced by CAR-T therapy. CAR-NK cells release relatively safe cytokines, rarely causing severe CRS or GVHD. They also possess diverse killing mechanisms that expand their recognition of tumor antigens, including those with missing or downregulated MHC class I molecules, which are typically not recognized by CAR-T cells. Furthermore, the abundant sources of CAR-NK cells facilitate the development of “off-the-shelf” products. However, CAR-NK therapy also faces limitations, such as poor transportation and infiltration in solid tumors and restricted antitumor activity and persistence in the immunosuppressive TME.

The low risk of rejection of NK cells allows for multiple sources such as the NK-92 cell line, peripheral blood mononuclear cells, umbilical cord blood, and induced progenitor stem cells (iPSC) [23,24]. Therefore, CAR-NK cells can be prepared in large quantities in advance and provide “off-the-shelf” products to patients, which are more convenient and less costly than the preparations of CAR-T cells (Figure 3). In summary, CAR-NK cells represent a highly promising cell type for solid tumor therapy following the advent of CAR-T cells.

### 3.3. Challenges and Therapies to Improve the Efficiency of CAR-NK

Although CAR-NK therapies demonstrate positive potential for the treatment of solid tumors, there are still some issues that must be considered before they can be truly applied in the clinic. Compared with the relatively complete CAR-T cell therapy, the CAR design of CAR-NK cells still has deficiencies, particularly in the design of the activation domain, which is the most critical. Currently, the CD28-CD3ζ combination has been proven to provide a strong activation signal [20], but further experiments are still needed to determine the optimal combination, sequence, and context of the activation domain [25]. In addition, cryopreservation of CAR-NK cells is unavoidable as it is almost impossible to administer fresh CAR-NK cells to patients. A technical challenge exists: NK cells are significantly more sensitive to freezing and thawing compared to T cells, and this process may substantially reduce their viability and cytotoxicity [26]. Key strategies addressing this point include co-incubation with IL-2, optimized cell thawing protocols [27], and DMSO-free cryopreservation techniques [28].

The infiltration density of endogenous NK cells in the microenvironment of solid tumors is significantly deficient, with numbers typically below 100 per unit area (mm^2^) [29], much less than the unit density of T cells. It is worth noting that the ability of NK cells to achieve adequate tumor infiltration is a key prerequisite for the establishment of an effective anti-tumor immune response, which not only has a direct impact on the effectiveness of immunotherapy but also has a significant correlation with the clinical prognosis of the patients [30].

NK cell trafficking and anticancer activity are affected by transcription factors (e.g., T-bet, promyelocytic leukemia zinc finger protein) [31,32] and TME-related metabolic profiles including, but not limited to, hypoxia, low pH, and elevated levels of adenosine, reactive oxygen species, and prostaglandin E_2_ [33]. It has been demonstrated that combining targeted chemokine receptors with CAR-NK cells enhances trafficking. NKG2D-CAR-NK cells overexpressing CXCR1 have shown enhanced in vivo solid tumor migration and infiltration in ovarian cancer xenograft mouse models [34]. In addition, overexpression of CXCR4 [35] and CCR7 [36] may also increase the chemotaxis of CAR-NK cells to solid tumor lesions. Natural killer cell engagers (NKCEs) may allow better targeting of CAR-NK cells to solid tumor sites, especially as multispecific NKCEs can promote stronger NK cell activation and binding specificity [37]. To overcome immunosuppressive TME, the main current approaches are to alter the metabolic composition of the tumor or to modify the gene expression program in immune cells. Examples include favorable regulation of TME with glycolysis inhibitors and LDH blockers [24]. An effective strategy for modifying genes is to block the TGF-β signaling pathway by CRISPR-Cas9 knockdown of the TGFβR2 gene in NK cells, which significantly enhances its tumor-killing ability in acute myeloid leukemia and glioblastoma [38].

Furthermore, tumor-associated macrophages (TAMs), cancer-associated fibroblasts, myeloid-derived suppressor cells, and regulatory T cells (Tregs), collectively impair NK cell-mediated cytotoxic activity, thereby promoting tumor immune evasion. Nevertheless, the interaction between CD4^+^ T cells and NK cells has been proven to be of considerable significance in anti-tumor responses. TME-resident T cells augment rituximab (RTX)-potentiated NK cell viability and ADCC, while synergistically targeting immune-evading MHC-I^low^ tumors [39]. Some interesting studies have also indicated that tumor-derived exosomes (TDE) play a key role in several aspects of NK cell dysfunction, but this role can be reversed by IL-15 [40,41].

Despite the lack of persistence of CAR-NK cells in vivo being relatively safe for patients, it also limits their therapeutic efficacy. Cytokines such as IL-2 and IL-15 are commonly used to enhance the viability and prolong the persistence of CAR-NK cell therapies. IL-15 exhibits a proliferative potency in NK-92 cells that is approximately 10 times greater than that of IL-2 [42]. Recently, Jianhua Luo et al. [43] engineered mesothelin-specific CAR-NK cells capable of secreting neoleukin-2/15 (Neo-2/15), which sustains enhanced IL-2 receptor signaling to upregulate c-Myc and nuclear respiratory factor 1 (NRF1) expression. This modification significantly potentiated CAR-NK cell cytotoxicity and prolonged their survival in solid tumor models [43].

To mitigate potential safety concerns, one strategy involves inserting suicide genes into CAR-modified effector cells, enabling rapid depletion of CAR-NK cells if needed [44]. Beyond such safety switches, researchers have developed strategies to refine CAR-mediated activation control. Logic-gated CARs enhance tumor-selective targeting, thereby reducing on-target/off-tumor toxicity and other adverse effects. Although this type of research on CAR-NK cells has been largely limited to hematologic tumors [45], it remains a very promising technology for application in the field of solid tumors.

## 4. CAR-M Therapies as a Rising Horizon in Immunotherapy

### 4.1. CAR-M in Solid Tumors

Macrophages are phagocytic and specialized antigen-presenting cells that play a wide range of roles in the clearance of pathogens and maintenance of tissue homeostasis. Macrophages constitute the predominant leukocyte population in solid tumors. Their prominent infiltration is attributed to the secretion of matrix metalloproteinases, which degrade key extracellular matrix components and basement membranes, enabling tumor stromal remodeling. Macrophages can be polarized into distinct functional states. The M1 phenotype, which is classically activated, exhibits pro-inflammatory and anti-tumorigenic functions. In contrast, the M2 phenotype, which is alternatively activated, is associated with pro-tumorigenic activities and tumor progression [46]. TAMs are a key component of the tumor microenvironment, and most are the M2 phenotype. Activated macrophages mediate multiple killing mechanisms (Figure 2), including cytotoxicity, phagocytosis, activation of adaptive anti-tumor immunity, and remodeling of the tumor microenvironment, and serve as a bridging point between innate and adaptive immunity [47].

Compared to CAR-T and CAR-NK therapies, CAR-M-based approaches for solid tumors remain in their infancy. The FDA has approved six CAR-M-based clinical trials. Currently, only Carisma’s first Phase I clinical trial (NCT04660929) has reported preliminary results, while the others are either in the early stages or have not yet been initiated. The data revealed that CAR-M infusion was well tolerated without dose-limiting toxicity (DLT) or serious side effects such as CRS and neurotoxicity [48]. Beyond this, several preclinical studies have demonstrated the potential efficacy of CAR-M in solid tumor species [49,50].

### 4.2. CAR Design for Macrophages: Boosting Phagocytosis and M1 Polarization

Unlike CAR-T and CAR-NK, the goal of optimal CAR-M design is to increase macrophage phagocytosis to eradicate tumors. First-generation CAR-M designs relied heavily on macrophages’ own ADCC and phagocytosis. Second and subsequent generations of CAR-M incorporate intracellular signaling domains, such as the TIR structural domain that can provide macrophages with polarized orthogonal signals [51]. Third-generation CAR-Ms under investigation are reprogrammed in vivo using non-viral vectors and are anticipated to significantly enhance the efficacy of anticancer therapies.

CAR-M doped with phagocytosis receptor intracellular domains can phagocytose antigen-specific target cells and exert anti-tumor effects. Morrissey and colleagues indicated that the phagocytosis domains Megf10 and FcRγ could effectively promote the phagocytosis of antigen-bearing beads by CAR cells [52]. As a prototypical intracellular structural domain of CAR-T, CD3ξ has also been shown to have comparable potential with FcRγ to promote CAR-M phagocytosis and has been widely used in CAR constructs [50]. In addition, CAR-M integrating the CD19 cytoplasmic structural domain can recruit PI3K, a key signaling pathway in phagocytosis, thereby increasing 3-fold the ability of CAR-M to phagocytose tumor cells [52].

Phagocytosis of TAMs has been elucidated to be a key determinant of tumor metastasis and is closely associated with TME [53]. Macrophage phagocytosis of cancer cells is significantly limited by phagocytic checkpoints, and cancer cells can evade macrophage clearance by overexpressing the “don’t eat me” signal. One of the earliest identified and most thoroughly studied phagocytic checkpoint axes is CD47-SIRPa, which acts directly through innate immunity [54]. Silent signal-regulated protein α (SIRP-α) was found to counteract the effects of CD47, thereby activating inflammatory pathways in CAR-M cells and inducing M1 polarization, which exhibited significant anti-HER2^+^ tumor effects [55] Tumor-associated macrophages (TAMs) also express Programmed Cell Death Protein 1 (PD-1), which binds to its cognate ligand, PD-L1, expressed on tumor cells, thereby contributing to cancer progression. Stefano Pierini’s team highlighted that combining CAR-M with an anti-PD-1 antibody in preclinical models significantly inhibited tumor progression, prolonged survival, and remodeled the tumor microenvironment in HER2-positive solid tumors with limited response to PD-1 monotherapy [56].

Because the M1 phenotype has significant antitumor effects and activation of M1 triggers adaptive antitumor immunity, driving and sustaining M1 polarization in CAR-M is a central goal of macrophage-based cancer therapy. It was found that transducing macrophages with the chimeric adenoviral vector Ad5f35 induced a durable M1 phenotype [50]. Another common approach is to incorporate into the intracellular region of the CAR receptors involved in the transduction of inflammatory pathways, such as toll-like receptor 4 (TLR4) and IFN-γ receptor [57]. CD3ζ-based CARs effectively induce Syk-dependent phagocytosis and targeted tumor cell killing in human macrophages without the involvement of soluble conditioners [50]. Even after long-term exposure to TME, phagocytosis was significantly enhanced, and M1-type macrophage polarization was promoted by designing tandem CD3ζ-TIR dual signaling structural domains in human iPSC-derived CAR-M [51].

### 4.3. Challenges and Perspectives for CAR-M Therapy

**Figure 4:**
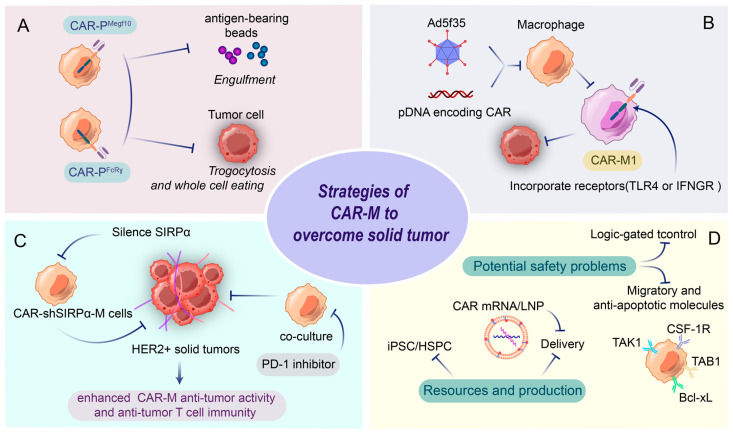
Challenges of CAR-M and engineering strategies to overcome solid tumors. CAR-M cells are engineered to enhance phagocytic activity and antitumor immunity through multiple strategies: (**A**) CAR constructs (e.g., CAR-PMegr10/PFcRγ) enable targeted engulfment of antigen-bearing tumor cells and beads, while trogocytosis facilitates whole-cell clearance. (**C**) Phagocytosis checkpoint. Silencing SIRPα disrupts the “don’t eat me” signal, amplifying tumor cell phagocytosis; additionally, combining CAR-M with PD-1 inhibitors augments T cell immunity in HER2+ solid tumors. (**B**) iPSC/HSPC-derived CAR-M, delivered via Ad5f35 or plasmid DNA, are polarized toward pro-inflammatory M1 phenotypes through TLR4/IFNGR signaling, enhancing cytokine secretion (e.g., IL-12) and stromal degradation. (**D**) Logic-gated controls and anti-apoptotic molecules (Bcl-xL, TAK1/TAB1) address safety risks, though challenges persist in migratory regulation and CSF-1R-mediated TME interactions. These integrated approaches position CAR-M as a versatile tool for overcoming solid tumor resistance.

Although preclinical and clinical trials of CAR-M have shown encouraging results, it still has significant limitations. First, macrophages have a limited ex vivo expansion capacity compared to T cells and NK cells. Given the patient tolerance and the number of macrophages in the body, there are studies demonstrating that CAR-M cells can be produced from alternative sources such as iPSC, human hematopoietic stem cells, umbilical cord blood [58], and macrophages obtained from ascites of cancer patients [59]. Secondly, due to the innate immune function of macrophages, it is difficult to transduce them with viruses, especially primary macrophages. Consequently, increasing research efforts have focused on non-viral delivery strategies, such as lipid nanoparticles (LNPs) for CAR mRNA delivery, to genetically modify both CAR-M and CAR-T cells [60]. These materials can be used as an alternative to viral vectors to deliver CAR genes, with the advantages of simplicity of production, ease of mass production, and lack of specific immune response. Unfortunately, although CAR-Ms have shown excellent antitumor effects in vitro, their in vivo effects are limited by delivery efficiency and targeting issues. Local delivery methods such as peritoneal injection have improved efficacy to a certain extent, but further breakthroughs are needed to target metastatic tumors. Moreover, the role and function of macrophages change dynamically in TME, so it is important to correlate the immunophenotypes of cancer patients before and after CAR-M treatment [61]. Finally, there may be potential safety issues, considering the long lifespan of macrophages in vivo. Various factors can induce apoptosis in macrophages, among which anti-apoptotic molecules such as Bcl-xL, Mcl-1, TAK1-binding protein 1, TGFβ-activated kinas activator, and TAK1-binding protein 2 are of great research value (Figure 4).

The development of next-generation CAR-Ms should prioritize three key pillars: molecular engineering, scalable manufacturing, and rational combination strategies to balance efficacy with safety. In order to enhance macrophage-specific phagocytosis and microenvironmental adaptation, CAR structural domains can be optimized and multifunctional modules can be integrated, including cytokines, phagocytic checkpoints, drug gating [62], and logic gating (AND/OR/NOT), among others. Meanwhile, combining CAR-M with CAR-T creates a synergistic innate–adaptive immune axis, while integration with conventional therapies may overcome tumor immunosuppression. While CAR-M holds transformative potential for solid tumors, its clinical success requires systematic optimization across biological discovery, production standardization, and multimodal therapeutic integration.

## 5. CAR-γδ T: Appealing Immune Effector for Clinical Cancer Immunotherapy

### 5.1. The Biology of γδ T Cells

γδ T cells are one of the earliest subpopulations of T cells to develop in the thymus and exhibit both innate and adaptive immunity [63]. γδ T cells account for 1–10% of circulating T cells in the peripheral blood of healthy adults, with Vδ1^+^ cells enriched in mucosal tissues, whereas Vδ2^+^ cells are most abundant in the blood and lymphoid organs [64]. The activation of γδ T cells is mediated through multiple pathways, including TCR signaling in combination with co-stimulatory cytokines (e.g., IL-15 and IL-18), as well as through engagement of NK cell receptors such as NKG2D. Members of the lactophilin and lactophilin-like (BTN/BTNL) family are key sensing factors for phosphoantigens in Vγ9δ2 cells, with BTN3A1 most widely recognized as an activator of the Vγ9Vδ2 subset of γδ T cells [65]. Activated γδ T cells have multiple killing mechanisms (Figure 2), including direct killing of target cells by a mechanism similar to that of αβ T cells and NK cells, as well as secretion of cytokines such as IL-17, IL-13, and IFN-γ, which indirectly contribute to the antitumor response [66]. In addition, Vδ2^+^ T cells can act as antigen-presenting cells to present antigen to αβ T cells with at least as much efficiency as DCs [67].

### 5.2. CAR-γδ T: Ideal Candidates for Cancer Immunotherapy

Several characteristics of CAR-γδ T cells render them a highly desirable alternative for oncology immunotherapy. These features include the lack of allogeneic reactivity, MHC-unrestricted recognition, broad-spectrum cancer cell recognition, ease of expansion both in vivo and in vitro, and the ability to present specialized antigens (Table 2).

The major γδ T cell subsets are Vδ1^+^ cells and Vδ2^+^ cells, with Vγ9Vδ2 cells being the most studied due to their stronger antitumor activity and the ability to be selectively expanded [68]. Solid tumors are highly heterogeneous, and conventional CAR-T suffers from tumor immune escape. However, the diversity of γδ T cell receptors makes them more sensitive to tumor-associated antigen (TAA) recognition and have a stronger tumor surveillance function. An intriguing study on gene expression has demonstrated that the infiltration of γδ T cells into tumors serves as a positive prognostic biomarker for many types of cancer [69]. M Girardi et al. have shown using mouse models that the epithelial localization of γδ T cells is conducive to the downregulation of epithelial malignancies [70]. It has been demonstrated that γδ T cells can provide protective immunosurveillance against spontaneously occurring mouse prostate cancer [71,72]. Similarly, preclinical studies in cancers such as breast, colorectal, ovarian, and renal cell carcinomas have demonstrated the antitumor activity of CAR-γδ T. Of high interest is the fact that γδ T cells preferentially destroy cancer cells and show hyporesponsiveness (if any) to healthy cells, as this suggests the therapeutic potential of γδ T cells [73].

γδ T cells have regulatory functions in TME. Numerous studies support this view; for example, a γδ1 T cell population that predominates among lymphocytes in infiltrating breast tumors has been demonstrated to suppress initial and effector T cell responses and IL-2 secretion, as well as inhibit dendritic cell maturation and function. Importantly, these immunosuppressive activities can be reversed by human TLR-8 ligands [74]. The tumor microenvironment can induce γδ T cells to secrete IL-17, which, in conjunction with neutrophils, promotes angiogenesis and metastasis [75]. Additionally, different subpopulations of γδ T cells (e.g., Vδ1 and Vγ9Vδ2) have functional plasticity, either exerting anti-tumor effects or shifting to an immunosuppressive phenotype in response to specific microenvironmental signals or artificial stimuli. This property suggests that the functional state of γδ T cells needs to be finely regulated to avoid potential bidirectional effects when utilizing them for immunotherapy.

Engineering αβ T cells with exogenous αβ TCRs faces a critical challenge: unintended pairing of transferred TCR chains with endogenous counterparts may form autoreactive TCR heterodimers, posing safety risks. However, it was demonstrated that transfer of the αβ TCR into γδ T cells does not generate neoreactive TCR heterodimers and has a more rapid response to target cells compared to conventional αβ T cells [76,77]. Moreover, it appears that γδ T cells can be generated on a clinical scale using an optimized expansion method [78]. Researchers have developed a novel regimen for selective expansion and differentiation of cytotoxic Vδ1^+^ (DOT) cells at the clinical level, which has demonstrated significant antitumor activity [79].

### 5.3. Challenges of CAR-γδ T Cells in Clinical Settings

Even though most current evidence suggests that CAR-γδ T cells are highly suitable for tumor immunotherapy, there are still some limitations to the clinical application of CAR-γδ T cells. γδ CAR-T cells generally have a lower clearance rate of tumor cells in vivo compared to αβ-CAR-T cells and therefore require multiple infusions and a large supply of γδ CAR-T cells. Part of the reason is the reduced persistence and cytotoxic activity of γδ CAR-T cells in the immunosuppressive tumor microenvironment, as well as the decreased antigen density due to antigen loss in target cells [80].

The tumor microenvironment can attenuate the antitumor activity of γδ T cells. In the tumor microenvironment, membrane-bound NKG2D ligands (e.g., MICA/MICB, ULBP) activate γδ T cells, but soluble NKG2D ligands inhibit NK and CD8^+^ T-cell functions by downregulating NKG2D. MICA-mediated sustained NKG2D signaling induces cellular depletion and inhibits cytotoxic cells expressing NKG2D [73]. Some metabolic features of TME such as oxygen partial pressure, reactive oxygen species, and cholesterol affect IFNγ and/or NKR expression [81]. Meanwhile, cytokines such as IL-2 and IL-7 are essential for T cell survival and proliferation, while IL-4 and IL-12 are important for determining the differentiation fate of T cells. Interestingly, IL-4 appears to have two aspects of action on suppressing the immune function of γδ T cells, including inhibition of TCR signaling as well as promotion of proliferation [82]. In addition, the inhibitory effect of Tregs on antitumor efficacy should also be considered [83]. In summary, the composition of the tumor microenvironment, the presence of immunosuppressive cytokines (IL-17 and IL-4), and Tregs should be evaluated before γδ T cell immunotherapy to minimize the risk of treatment failure in the clinical setting.

**Figure 5:**
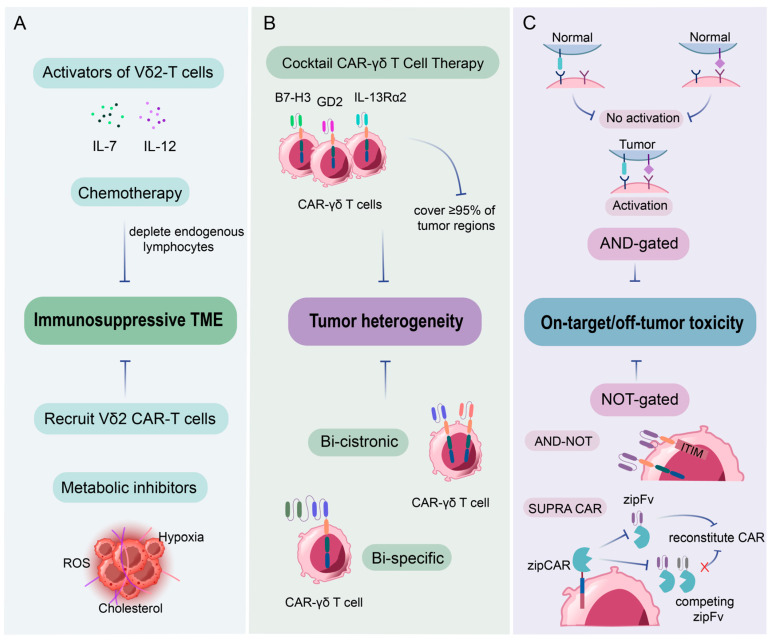
CAR-γδ T: challenges and strategies towards solid tumors. CAR-γδ T cell therapy employs combinatorial approaches towards solid tumors: (**A**) Immunosuppressive TME: activation of Vδ2 T cells via IL-7/IL-12 or chemotherapy-induced lymphodepletion enhances tumor targeting, while metabolic inhibitors (ROS, cholesterol) counteract immunosuppressive TME. (**B**) To solve the antigen escape problems, multi-antigen “cocktail” (B7-H3, GD2, IL-13Ra2, “Bi-cistronic”, “Bi-specific”, and “OR-gated”) strategies are used. (**C**) Logic-gated systems (AND/NOT) restrict activation to tumor-specific dual-antigen profiles, minimizing on-target/off-tumor toxicity. Innovations like bi-cistronic/bispecific CARs and SUPRA CAR systems further refine specificity and safety. These strategies collectively enhance precision and adaptability, positioning CAR-γδ T cells as a robust platform for heterogeneous solid tumors.

Many methods have been developed to optimize CAR-γδ T immunotherapy. Examples include the use of bispecific antibodies to enhance the cytotoxic activity and tumor-targeting of γδ T cells, but their main limitation is the complex manufacturing process. To control the on-target/off-tumor toxicity of CAR-γδ T cells, Fisher et al. designed a GD2-targeted CAR-γδ T cell in which γδ-T cell activation signals 1 and 2 are provided by separate receptors [84]. Clinical translation of γδ T cell therapies will require systematic resolution of multiple key issues, and it is important to identify appropriate patients and healthy donors and to develop standardized monitoring guidelines. It is also important to address disease prognosis and relapse and to determine whether to choose monotherapy or combination therapy [85] (Figure 5). To further realize the potential of γδT cell therapy, we look forward to more specific biomolecular studies, more clinical trial data, and a more optimized industrial chain.

## 6. Exploring Combination Therapies in CAR Cell Therapy

To further enhance the efficacy of CAR cell therapy in treating tumors, the development of novel CAR cells is being complemented by the continuous emergence of new combination therapies involving CAR cells. The functional characteristics of these combination therapies are theoretically capable of promoting the function of CAR cells from different perspectives.

Radiotherapy and chemotherapy, both conventional tumor treatment methods, have been shown to have immunomodulatory functions. Radiotherapy exerts cytotoxic effects by directly or indirectly damaging cellular DNA via the release of high-energy radiation. The discovery of the “distant effect” (the phenomenon where non-irradiated tumor lesions shrink while the irradiated tumor lesion is treated) [86] has also revealed its anti-tumor immune effects. Radiotherapy has been proposed as an adjuvant to several immunotherapies, including ICLs, CAR cell therapy, and tumor vaccines. There are currently several clinical and preclinical studies underway, with clinical studies focusing on the use of this combination therapy in hematologic tumors. Preclinical studies using mouse models have shown that local irradiation improves the efficacy of CAR-T cell therapy in solid tumors, such as glioma [87] and pancreatic cancer [88]. In addition, the combination of CAR-NK cell therapy and radiotherapy has shown improved anti-tumor activity in various tumor models [89].

The combination of chemotherapy and CAR cell therapy is very flexible. Chemotherapy controls disease and reduces tumor burden during the CAR treatment gap. It also serves as a pretreatment to create a host immune environment for infused cells that is most conducive to anti-tumor function [90]. Moreover, studies have demonstrated that certain chemotherapeutic agents, including cyclophosphamide, gemcitabine, doxorubicin, and paclitaxel, may synergize with CAR cell therapy. Cyclophosphamide has been demonstrated to deplete Tregs and promote T cell recovery [91], and it can also mitigate immune effector cell-associated neurotoxicity syndrome related to CAR-T cell therapy [92]. As with radiotherapy, the inherent cytotoxic effects of chemotherapy have the potential to damage CAR cells and other immune cells. This is a critical consideration that must be taken into account when applying chemotherapy.

As previously stated, immune checkpoints are a group of signaling pathway molecules that are widely distributed in solid tumors and can regulate the persistence of immune responses. Mainstream combination approaches include the use of exogenous ICLs and the expression of antibodies by CAR cells through CAR editing. Persistent stimulation of PD1 in the tumor microenvironment can directly result in T-cell depletion, and CD8+ T-cell depletion has been directly correlated with the abundance of tumor-associated macrophages. A study by Stefano Pierini’s team demonstrated that the combination of CAR-M and anti-PD-1 antibodies significantly inhibited tumor progression, particularly in HER2-positive solid tumors [56]. In a subcutaneous CRC mouse model, the administration of CAR-T cells that secrete PD-1-TREM2 scFv was found to result in the more efficient and persistent elimination of tumors [93]. However, it is imperative to acknowledge the critical function of PD1 in the development and maturation of immune cells. Consequently, the utilization of PD1 may potentially mitigate the anti-tumor efficacy of CAR cells [94]. Despite the numerous preclinical studies that have demonstrated the efficacy of ICLs and CAR cell immunotherapy, further clinical research is necessary to assess their safety and determine optimal utilization strategies.

The utilization of viruses in cancer therapy has a long history, dating back to the early 19th century. This approach is now referred to as “oncolytic viruses” (OVs). Oncolytic viruses have been shown to elicit systemic innate and tumor-specific adaptive immune responses, thereby suppressing tumors by preferentially replicating in tumor cells and directly killing infected tumor cells [95]. The primary advantage of combining OVs with CAR cell therapy for the treatment of solid tumors is that OVs enhance the immune activity of the TME [96]. This enhancement significantly improves the delivery of CAR cells into solid tumors and promotes the expansion and maintenance of CAR cell function [97]. A preclinical study targeting pancreatic cancer demonstrated that the combination of mesothelin-targeted chimeric antigen receptor T cells and cytokine-armed oncolytic adenoviruses improved pancreatic cancer treatment by overcoming T-cell dysfunction and tumor heterogeneity in target antigen expression [98]. Porter et al. [99] developed a binary oncolytic helper adenovirus expressing a bispecific T-cell engager (BiTE), IL-12, and an anti-PD-L1 antibody. When combined with CAR cells, this virus significantly enhances tumor control and survival in both HER2-positive and HER2-negative cancer models [99]. Preclinical studies conducted on solid tumors have yielded favorable outcomes, including head and neck cancer and neuroblastoma [100].

Different CAR cells can also be combined to enhance each other’s efficacy. One strategy is to combine CAR-T cells with different structures and also to use CAR-T cells that target different antigens on tumor cells [101]. Given the high infiltration of macrophages in solid tumors and their ability to stimulate T cells, combining CAR-M and CAR-T cells represents a logical approach to optimize antitumor effectiveness, as demonstrated in mouse models of glioma [102,103].

In addition, there are many other combination therapies that hold significant research value. Immune modulators, metabolic inhibitors, and cytokines, as mentioned earlier, can all be combined with CAR cell therapy [104,105], which will not be further elaborated here. In summary, the combination of CAR cell immunotherapy with other therapeutic modalities is likely to be one of the key breakthroughs in future cancer treatment.

## 7. Conclusions

The therapeutic field and potential of CAR therapies are expanding rapidly and steadily, from autologous infusion to allogeneic therapies, from hematological tumors to solid tumors, and from the field of cancer to other diseases. This is a clear indication of the important place they will occupy in the future of immunotherapy. Despite the theoretical feasibility and positive preclinical and clinical trial results of using NK cells, macrophages, and γδ T replacement T cells in the treatment of solid tumors, there are still objective limitations to each of them (as discussed earlier in this article). Take CAR-NK cell for an example, its anti-tumor activity is highly dependent on the impact of TME, which really restricts the use of CAR-NK. While many design strategies for remodeling the tumor microenvironment have also been shown to be effective in preclinical models, there are difficulties in translating them into clinical applications, including systemic infusion of NK cell-activating factors and problems with frozen storage of NK cells.

Novel CAR designs and combination therapies are emerging, showing great promise in improving the efficacy of CAR therapies in solid tumors. These strategies incorporate the characteristics of solid tumors and the immunological profile of each cell to minimize the risks and disadvantages inherent to each specific cell population while maximizing efficacy and treatment durability. CAR therapy in combination with immune checkpoint inhibitors, radiotherapy [106], and chemotherapy is very promising. The future of immunotherapy for solid tumors is undoubtedly bright, thanks to the development of novel CAR cells and the ongoing clinical trials that are shaping the field.

## Figures and Tables

**Table 1:** The key milestones of CAR-T cell development.

Year	Key Events	Structure Features	Meanings
1988	TILs used to treat metastatic cancer		Enhanced tumor-specific immune response against metastatic cancer
1993	A chimeric gene developed to provide effector lymphocytes with antibody-type recognition [4]	CAR consists of the extracellular domain, the transmembrane domain, and the intracellular signal transduction domain (CD3ζ chain)	1st generation CAR-T prototype described
1998	CD3/CD28 beads induce ex vivo expansion of human T cell	A co-stimulation domain (CD 28) added to CAR	2nd generation CAR-T
2004	4-1BB signaling capacity provoked potent cytotoxicity against ALL [5]	4-1BB used as the co-stimulation domain	2nd generation CAR-T
2008	GD2- CAR-T cells showed antitumor activity and safety in neuroblastoma [6]	CAR is directed to the diasialoganglioside GD2	Virus-specific CTLs can be modified to function as tumor-directed effector cells
2010	CD28 endodomain showed remarking enhanced expansion and persistence in lymphoma patients [7]	Combining two co-stimulatory molecules (CD28 ICOS, 4-1BB, OX40, and CD27)	3rd generation CAR-T arise
2011	The remission of leukemia patients is ongoing after CAR-T treatment (2nd generation CAR-T) [8]		Promise of 2nd generation CAR-T in leukemia treatment.
2012	Decade-long clinical trials proved the safety and function of 1st generation CAR-T for HIV [9]; engineered CAR-T cells deliver inducible IL-12 to combat cancer	Engineer CAR-T with IL-15/IL-12/IL-18 and a suicide gene	Paved the way for CAR-T therapy in HIV treatment; 4th generation CAR-T arise
2017	FDA approval CD19 CAR-T therapy for leukemia		Marked a major breakthrough in cancer treatment, especially offering new hope for patients with relapsed or refractory leukemia
2021	FDA approval of: abecma for multiple myeloma; CAR-T for autoimmune disease (SLE)		The achievement of CAR-T in multiple myeloma treatment and the expansion of CAR-T therapy into new disease areas
2023	Mutated c-KIT added to CAR-T		Focus on solid tumors

**Table 2:** Comparison of the four novel CAR cell therapies.

	CAR-T	CAR-NK	CAR-M	CAR-γδ T
Mechanisms of cell killing	CAR-dependent T-mediated cell killing; cytokine release; antigen presentation; TME remodeling	CAR-dependent NK -mediated cell killing; innate cytotoxicity; cytokine release; ADCC	CAR-dependent phagocytosis, cytotoxicity, pro-inflammatory secretion, antigen presentation, TME remodeling	CAR-dependent cell killing, indirect antitumor contribution, antigen presentation, direct cytotoxicity, cytokine release, ADCC
Cellular sources	Autologous, MHC-matched allogeneic, T cell lines	Autologous, non-MHC-matched allogeneic, NK cell lines	Autologous (iPSCs and cell lines are used in preclinical studies)	Autologous or allogeneic γδ T cell lines (peripheral blood, iPSC-derived, tissue-resident γδ T cells)
In vitro expansion	Effectively expanded in vivo using optimized culture conditions and cytokines	Efficiently expanded in vitro with specific cytokines	Limited ability to expand, but alternative sources (iPSC and cell lines) can be used	Readily expandable in vitro (especially Vγ9Vδ2)
Production	Time-consuming and costly	“Off-the-shelf” products	Time-consuming, but with potential for “off-the-shelf”, low-cost, and standardized products	Potentially “off-the-shelf” products
Antigen recognition	Specific antigen recognition via CAR	Specific antigen recognition via CAR, with additional innate cytotoxicity	Specific antigen recognition via CAR, with phagocytic and antigen-presenting capabilities	Specific antigen recognition via CAR, with additional innate TCR-mediated recognition
Infiltration in solid tumors	Poor	Poor	Abundant	Moderate
Persistence	Long-term	Short-term	Moderate to long-term (depends on the immune environment)	Lack of clear persistence data
Toxicities	Common and serious CRS/neurotoxicity; GVHD; on-target/off-tumor toxicity	Less common and serious CRS/neurotoxicity; do not cause GVHD	Lack of clear clinical data	No formal study comparing the toxicities so far; serious CRS has not been reported in preclinical studies so far
Clinical status	Proven efficacy in hematologic malignancies; six CAR-T therapies approved by the FDA	Limited clinical trials and no approved therapy. Three trials have been completed; one trial has been published	Still at an early stage; a first-in-human (phase 1) multicenter clinical trial has been published	No approved therapy; several early-phase trials are ongoing
Advantages	Prolonged durability; proved strong efficiency in hematologic malignancies; high antigen specificity	Abundant cell sources; providing “Off-the-shelf” products; low toxicity; multiple cell killing mechanisms	Abundant infiltration in solid tumor; various alternative sources; M1 macrophages in TME have pro-inflammatory and anti-tumor effects; phagocytosis of TAMs is key for tumor metastasis closely related to TME	Multiple killing mechanisms; broad-spectrum cancer cell recognition; ease of expansion; specialized antigens presenting
Disadvantages	Poor tumor trafficking and infiltration; lack of antigen heterogeneity; limited persistence in the immunosuppressive TME; serious toxicities; high cost of manufacturing	Very poor tumor infiltration; low CAR transduction efficiency; short-term persistence; limited ex vivo expansion	Difficult to transduce with virus; the phenotypes of macrophages change dynamically in TME	Low clearance rate of tumor cells in vivo; immunosuppressive TME has a significant impact on persistence and cytotoxic activity
